# Outcome of Surgery as Part of Palliative Care of Patients with Symptomatic Advanced or Metastatic Extra-Abdominal High-Grade Soft Tissue Sarcoma

**DOI:** 10.1089/pmr.2022.0063

**Published:** 2023-03-17

**Authors:** Farhad Farzaliyev, Hans-Ulrich Steinau, Andrej Ring, Rainer Hamacher, Tobias Thiel, Henrik Lauer, Lars Erik Podleska

**Affiliations:** ^1^ Department of Hand, Plastic, Reconstructive and Burn Surgery, BG Klinik, Eberhard Karls University, Tuebingen, Germany.; ^2^ Department of Tumor Orthopedics and Sarcoma Surgery, University Hospital Essen, Essen, Germany.; ^3^ Department of Plastic and Reconstructive Surgery, St. Rochus Hospital, Castrop-Rauxel, Germany.; ^4^ Department of Medical Oncology, Sarcoma Center, West German Cancer Center, University Hospital Essen, Essen, Germany.

**Keywords:** advanced soft tissue sarcoma, palliative plastic surgery, retrospective study, sarcoma resection

## Abstract

**Background:**

The modern multimodal treatment of malignant tumors has increased disease-specific survival and decreased the burden of tumor-associated complications. The main focus of palliative surgery is not based primarily on quantitative success parameters of tumor response but is instead mainly on the question of quality of life.

**Aim:**

The current study was conducted to analyze the clinical and oncological outcomes of palliative patients with soft tissue sarcoma.

**Design:**

Of 309 patients with extra-abdominal high-grade soft tissue sarcoma treated between August 2012 and December 2014, our retrospective analysis revealed 33 palliative patients for this study. All patients were evaluated and managed by a multidisciplinary team with expertise and experience in sarcoma treatment. The survival analysis was made using the Kaplan–Meier method.

**Results:**

The main sarcoma symptoms were pain (27.3%) and ulcerated tumors or shortly before ulceration (24.2%). Thirteen patients (39.4%) were operated on with negative margins, 15 (45.5%) with positive margins, 2 with tumor debulking (6.1%), and 3 patients (9.1%) were treated only with palliative hyperthermic isolated limb perfusion. Ten pedicle flaps were performed after sarcoma resection. The median operation time was 85 minutes (range, 37–216 minutes). The median hospitalization stay was 9.5 days (range, 3–27 days). No patients died during hospitalization. Twelve-month disease-free survival was 48.5% (95% confidence interval: 45.4–51.6).

**Conclusions:**

Palliative surgery of metastatic or advanced soft tissue sarcoma can improve the wound care and quality of life. Closed noninfected wounds enable further treatment options, such as chemotherapy, immunotherapy, and radiotherapy. This surgery should be considered during the discussion on interdisciplinary tumor boards.

## Highlights

- Modern multimodal treatment of sarcomas has increased disease-specific survival and relief from tumor-associated complications.
- Palliative and plastic-reconstructive surgery as a part of multimodal therapy can decrease the burden of tumor-associated complications.
- Palliative plastic-reconstructive surgery should be considered during the discussion on interdisciplinary tumor boards.

## Introduction

The term “palliation” defines the indications in oncological surgery for incurably ill patients with locoregional or distant metastases. The patient's condition is severely impaired by rapid growth, infiltration, inflammation, or ulceration of the primary tumor, local recurrence, or metastasis in soft tissue.^1^

Soft tissue sarcomas can often grow locally into monstrous tumors, even without regional and distant metastases. They can therefore disable and burden patients by restricting their ability to move, by causing weeping and fetid wound surfaces, and resulting in tumor decay cavities, as well as their frequently frightening appearance. Such tumors are highly probable to require palliative plastic-reconstructive surgery in the terminal stage.^2,3^

The modern multimodal treatment of malignant tumors has increased disease-specific survival and relief from tumor-associated complications. The goal of palliative surgery is no longer principally based on quantitative success parameters of tumor response but primarily includes the question of quality of life.^4,5^

Only limited data are available on the specific challenges of palliative surgery in extra-abdominal soft tissue sarcoma.

A previous study showed the impact of specialized palliative care interventions with a multidisciplinary approach on symptom relief and quality of life in patients with advanced soft tissue sarcoma.^6^ This study aims to describe the results of palliative surgery as part of this specialized palliative care intervention.

## Materials and Methods

Of 309 patients with extra-abdominal high-grade soft tissue sarcoma treated between August 2012 and December 2014, our retrospective analysis revealed 33 eligible patients for our study.

Patients were included if they had:

1. Non-operable primary or recurrent tumor;
2. Operable primary or recurrent tumor with evidence of metastatic sarcoma;
3. Metastasis of sarcoma in soft tissue.

A vote of approval for this study was obtained from the ethics committee (20-9398-BO). Non-operable primary/recurrent tumors, as well as metastasis in soft tissue, were diagnosed with magnetic resonance imaging (MRI). In most cases, the diagnosis was confirmed with Tru-Cut^®^ biopsy. In the case of multiple soft tissue lesions with radiographic signs of metastasis, a biopsy was not performed. Thoracic and abdominal metastases were revealed with computer tomography scans. Typing and grading of tumors were determined according to the WHO and TNM classifications.^7^ All fibroblastic and myofibroblastic high-grade sarcomas were defined as myxo-/fibrosarcoma, and sarcoma subgroups smaller than three were included in group “others.” Tumors with histopathological grading G2 and G3 were classified as high-grade soft tissue sarcoma.

All patients were evaluated and managed by a multidisciplinary team with expertise and experience in sarcoma treatment. Data were collected from clinical correspondence and hospital notes.

### Palliative treatment

The main goal of the surgical therapy was:

1. Resection of operable primary/recurrent tumors or metastasis in soft tissue. Limb salvage was prioritized over negative margin or;
2. Symptomatic treatment of sarcoma disease, such as pain and fungating tumors, to improve quality of life.
3. Palliative isolated limb perfusion with tumor necrosis factor alpha and melphalan. This is a surgical procedure during which the affected limb is treated with localized hyperthermic chemotherapy: 1 mg tumor necrosis factor alpha (Beromun, Boehringer Ingelheim, Ingelheim, Germany) and weight adapted doses of melphalan.^8^

For the patients with defects after resection of large ulcerating tumors, local skin or pedicle musculocutaneous flaps were performed after resection. After surgery, all patients were presented in multidisciplinary tumor boards to determine further palliative therapy. Radiation therapy and chemotherapy were administered according to the protocols for palliative soft tissue sarcoma according to the Guidelines of European Society of Medical Oncology and National Comprehensive Cancer Network for soft tissue sarcoma (www.nccn.org/professionals/physician_gls #soft-tissue-sarcoma).^9^

### Statistical analysis

Follow-up was performed five years after the treatment of the last patient. Information about patients was obtained from hospital notes or the family physician. The primary endpoint was determined as disease-free survival, measured from the date of surgical treatment to time of death or the last day of follow-up. Periods at risk of death were defined in months for each patient. Statistical analysis was performed with SPSS (Statistical Package for the Social Sciences) software, version 23.0.

## Results

### Patients and treatment characteristics

Table 1 summarizes the patient's common demographic and clinical characteristics. Sixteen patients (48.5%) had operable primary/recurrent tumors with distant metastases at presentation. Ten patients (30.3%) had non-operable primary or recurrent tumors without metastatic disease, most commonly located on the trunk. Of these, only one hemiplegic patient had a non-operable recurrent tumor on the contralateral arm. The patient denied amputation because she used this arm for self-care. She was treated only with hyperthermic isolated limb perfusion with tumor necrosis factor-alpha and melphalan. Seven patients (21.2%) were treated due to metastasis of sarcoma in soft tissue.

**Table 1.** Patient's Common Demographic and Clinical Characteristics

Parameter	Patients, ***n*** (%)
Median age (range), years	57 (22–85)
Sex	
Male	18 (54.5)
Female	15 (45.5)
Localization of primary/recurrent tumor or soft tissue metastasis	
Trunk (including abdominal, thoracic wall, gluteal, pelvis, groin, and axilla)	17 (51.5)
Extremity	16 (48.5)
Histological findings	
USTS	12 (36.4)
Myxo-/fibrosarcoma	5 (15.2)
Others	16 (48.4)
Presentation	
Operable primary/recurrent tumors with metastases	16 (48.5)
Non-operable primary and recurrent tumors without metastases	10 (30.3)
Metastases of sarcoma in soft tissue	7 (21.2)
No. of patients	33

USTS, undifferentiated soft tissue sarcoma.

The main symptoms of sarcoma manifestation were pain (27.3%) and ulcerating tumors or tumors shortly before ulceration (24.2%).

**FIG. 1.**
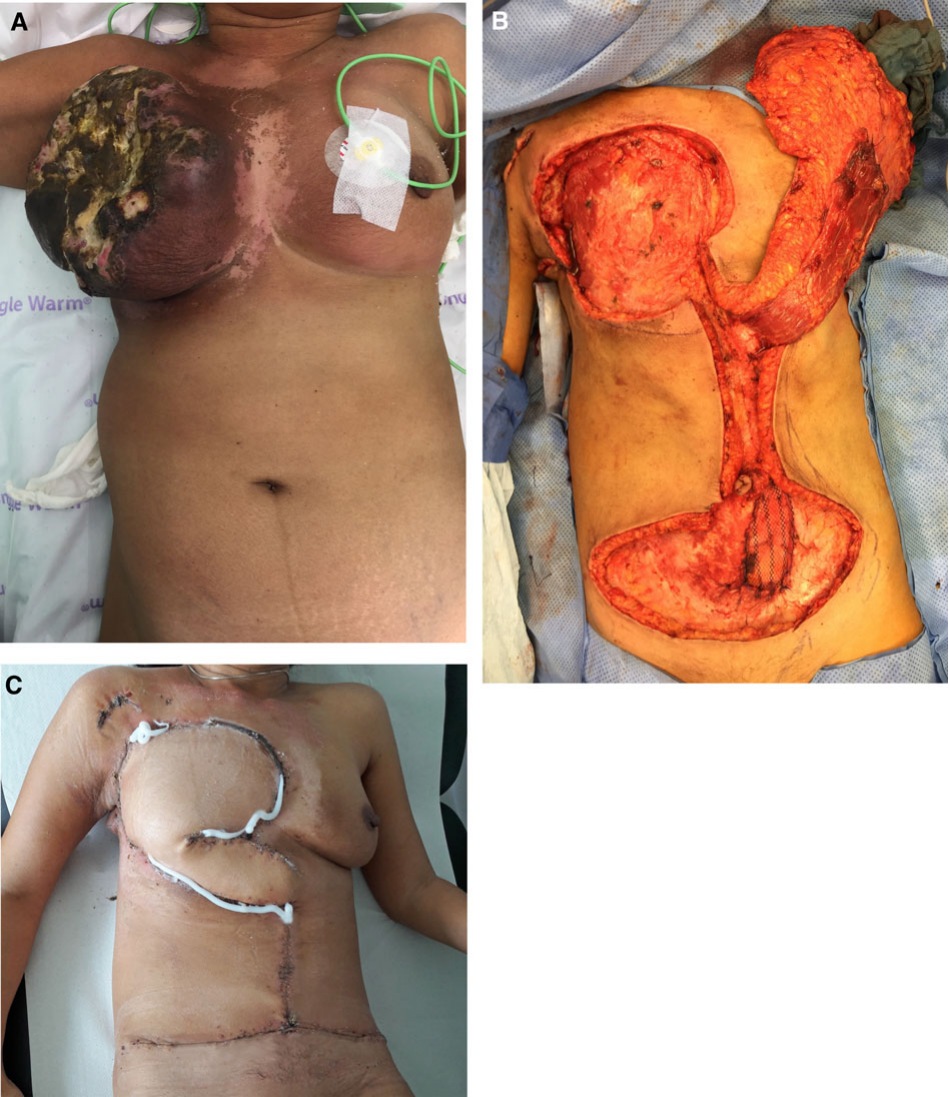
**(A)** Fungating recurrence of high-grade metastatic sarcoma of the right breast. Due to recurrent bleeding of the rapidly growing tumor, emergency radiotherapy with 36 Gy was performed in other hospital. **(B)** After discussing the case in our interdisciplinary tumor board, sarcoma resection and subsequent reconstruction of the defect (∼400 cm^2^) using a combined pedicled vertical and transverse rectus abdominis musculocutaneous flap were performed. Final pathological findings showed negative resection margins with a minimum distance of 1 mm to the deep. The patient was discharged from the hospital after 13 days with good wound conditions. **(C)** Follow-up after four weeks. Adjuvant palliative chemotherapy was performed. The patient died 13 months after surgery due to metastatic disease without local recurrence.

Thirteen patients (39.4%) were operated with negative margins, 15 (45.5%) with positive margins, 2 with tumor debulking (6.1%), and 3 patients (9.1%) were treated only with palliative hyperthermic isolated limb perfusion. No amputation was performed. Ten pedicle flaps were performed after sarcoma resection: nine on the trunk and one on extremity. The median operation time was 85 minutes (range, 37–216 minutes). Two patients were treated with more than one operation. One patient had a large infected fungating tumor of the breast wall (Fig. 1A). Defect closure with a pedicled transversal/vertical rectus abdominal flap from contralateral side was performed on this patient five days after tumor resection (Fig. 1B). The second patient required a second surgery due to wound infection. The median hospitalization stay was 9.5 days (range, 3–27 days). No patient died during hospitalization. Patients with fungating tumors no longer suffered from malodorous wounds and were able to re-establish social contacts.

According to the decision of the multidisciplinary tumor board, 12 patients (36.4%) were treated with neoadjuvant chemotherapy, 5 (15.2. %) with adjuvant, 5 (15.2%) with neoadjuvant and adjuvant, and 11 (33.3%) patients with no chemotherapy. Only 10 patients were treated with radiotherapy (33.3%). During the follow-up, in nine patients (27.3%), tumor recurrence was identified by MRI. No further exulceration developed in this group of patients.

### Disease-free survival

**FIG. 2.**
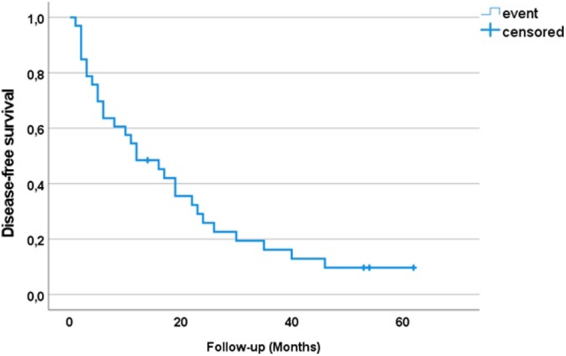
Kaplan–Meier plot of disease-free survival of patients with symptomatic advanced or metastatic extra-abdominal soft tissue sarcoma treated with multimodal therapy (*N* = 33 patients).

At the time of follow-up, 29 (87.9%) patients died of disease progression and 3 patients (9.1%) were alive. Information about one patient (3%) is not available. At the time of analysis, the median follow-up for disease-specific survival was 12 months (interquartile range 1–62). Twelve-month disease-specific survival was 48.5% (95% confidence interval: 45.4–51.6) (Fig. 2).

## Discussion

The surgical resection of extra-abdominal high-grade soft tissue sarcoma with oncologically appropriate margins is an important part of multidisciplinary team management, including plastic and reconstructive surgeons.^10^ However, in the case of locally advanced or incurable tumors, palliative surgical interventions are becoming increasingly significant due to modern oncological treatment and palliative care that improve patient's overall survival up to 12–18 months.^11–13^ Similar results were observed in our study.

The data according to the surgical palliation of high-grade soft tissue sarcoma are limited. Furthermore, it is challenging to correctly assess the situation and choose the correct indication for treatment. The indication for tumor resection by metastatic disease or advanced soft tissue sarcomas is based on the consideration that the procedure is designed to improve the patient's present condition or prevent possible symptomatic problems due to the tumor's growth. The predominant indication for admission to the palliative care unit was pain (*n* = 23, 67% of patients), followed by weakness (*n* = 15, 28%) and symptomatic tumor progression (*n* = 15, 28%).^6^

According to the recommendations for curative tumor therapy, palliative surgery should primarily aim to complete the removal of the tumor. Otherwise, particularly in the case of fast-growing tumors, the recurrence-free interval can be shortened, and another intervention is necessary. Resections with microscopically positive margins can be planned with additional adjuvant radiotherapy and chemotherapy. However, resection with macroscopically positive margins or “debulking” should only be reserved for inoperable tumors and advanced clinical conditions. The surgical methods are primarily influenced by the location of the lesion, the exposed structures, existing damage, accompanying additional therapies, and the patient's condition. If an operation is performed in an area already irradiated, preference should be given to muscles or fascial flaps with good blood flow.

Superficial, ulcerated, and infected soft tissue sarcomas can cause physical and psychological torment for the patient due to pain, erosion bleeding, foul odor, and severe disturbance of the body image, and thereby often drive them into isolation. Foul smelling wounds and ulcerations are one physical reason to wish to die. The patients feel ashamed about their ulcerating and strong-smelling tumors.^14^

In general, ulcerated tumors or repeated local recurrences are experienced by the patient as a visible daily experienced stigma of their life-ending disease. As a result of soft tissue plastic measures, a psychological consolidation can be expected in addition to functional improvement. In addition, the patient benefits from being released early into his usual social environment for the last few months of his life instead of having to endure repeated changes of bandages, removal of necrosis, antiseptic rinses, excruciating pain, and disturbing wound secretion.

Another essential factor that makes plastic surgery a valid option is that, unlike hospital staff, ulceration with an odor does not mean “normality” for close relatives but instead has a repulsive effect. This significantly influences personal attention, personal hygiene, and contacts. The restoration of an intact soft tissue coat offers further advantages, especially in care in a familiar environment at home. While open wounds often indicate hospitalization or require regularly trained nurses, primary care is much easier to provide without these issues. The dependence of a terminally ill patient on specialized facilities should therefore be delayed for as long as possible.

In the case of these tumors, the intervention will hardly affect the natural course of the disease. Nevertheless, self-sufficiency, hygiene, maintaining social contacts, and, last but not least, basic needs such as preventing odor nuisance and, finally, care can also be significantly improved under hospice conditions.^15–17^

Some authors suggested using vacuum-assisted closure with conventional foams after resection of malodorous tumors as alternative methods at the end of a patient's life. A disadvantage of this method is that negative pressure wound therapy dressing should be changed every five to seven days.^18^

Plastic-reconstructive procedures can solve the problem with the significant defects after resection of large tumors and restore lost function. Local, pedicled, and even free microsurgical flaps can be used for reconstruction as demonstrated in Figure 1C. The main principle remains: surgery is planned with the risk-to-benefit ratio being carefully considered.^19^ Reconstruction with non-radiated well-perfused flaps can drastically shorten the interval to the subsequent chemotherapy or radiation and prevent infection complications in deeper structures, such as bones and tendons.^20,21^

In the case of palliative operations, there must be an adequate relationship between the size of the procedure and the benefit to the patient. In addition, a low level of pain caused by the procedure, the shortest possible inpatient stay, and rapid rehabilitation should be required. The assessment of this situation can only be carried out in an interdisciplinary tumor board to weigh up the various options, such as radiation, chemotherapy, or isolated limb perfusion.^3^

This publication has three significant limitations that could be addressed in future research. First, limited patient cohorts do not allow a comprehensive assessment of oncological results. Second is the heterogeneity of the reasons for the need for palliative surgery and, based on this, different methods of treatment. Third, a lack in assessing the quality of life before and after surgery. Unfortunately, it cannot be done with this patient group.

## Conclusions

As a result of constantly evolving multimodal therapy, which has improved survival rates, palliative surgery serves several purposes. Tumor resection or “Debulking” and plastic reconstructive procedures can improve the wound care, infection control, psychological consolidation, quality of life, and maintenance and upkeep of social contacts. Closed, not infected wounds enable further treatment options, such as chemotherapy, immunotherapy, and radiotherapy. This type of surgery should be considered during the discussion on interdisciplinary tumor boards.

## Data Availability

The data that support the findings of this study are available on request from the corresponding author.

## Abbreviation Used

MRI: magnetic resonance imaging
